# Commentary on Manne et al.: Using the Interdependence Model to Understand Spousal Influence on Colorectal Cancer Screening Intentions: a Structural Equation Model

**DOI:** 10.1007/s12160-012-9350-0

**Published:** 2012-02-25

**Authors:** Mariët Hagedoorn

**Affiliations:** Department of Health Sciences, Health Psychology Section, University of Groningen, University Medical Center Groningen, Antonius Deusinglaan 1, 9713 AV Groningen, The Netherlands

Preventive colorectal cancer screening can significantly reduce mortality, but unfortunately, the participation in screening is still relatively low [1]. Although existing individual level models (e.g., health belief model) have increased our understanding of colorectal cancer screening behavior, the empirical evidence for the associations between many individual level factors and screening behavior is still limited [2]. Manne and colleagues [3] argued convincingly that a greater understanding of the role of one's significant other in screening intentions might help improve screening rates. The dyadic approach posits that members of couples are interdependent and influence each other's attitudes, behaviors, and health outcomes [cf. 4]. Put differently, participation in screening is not based only on individual factors but also on the interaction with one's intimate partner. In line with this idea, they found agreement with respect to screening practices in 65% of the couples.

The notion of interdependency of couple members in terms of behavior is not new, but to my knowledge, the study of Manne et al. is the first that actually investigated screening intentions at a dyadic level. The findings are promising in that they support the importance of spousal attitudes and communication within couples in the decision to participate in screening. The results showed that participants who felt that screening was important for both their partner and themselves were more likely to discuss the issue with their partner. Furthermore, couples who discussed the issue more often reported stronger screening intentions.

The findings suggest that a focus on couples instead of individuals may be an interesting strategy to increase screening rates, but they also raise important questions for further research. Manne et al. asked both members of the couple how often they had discussed screening, but did not look into the communication process. This process may vary depending on whether the partners have similar or different attitudes. Prior research suggests that close partners who confront attitude discrepancies are motivated to correct the imbalance by changing their attitudes, especially if the issue is central to the partner but not to themselves [5]. The more central the issue is to oneself, the greater pressure one exerts on the partner to change. The magnitude of the discrepancy, the centrality of the screening issue, and the tactics used may be key in the communication process, and hence couples' screening intentions.

Another issue is the study's focus on intentions. A previous review has shown that almost half of the participants with positive intentions failed to perform the intended behavior [6]. Furthermore, past behavior often is more predictive of future behavior than intentions. There is some evidence though that for infrequent behavior for which habits have not been formed deliberative reasoning may be a relatively strong predictor of actual behavior [7]. Considering that all couples in the analyses had been non-adherent with screening recommendations in the past, and it is unknown whether they had given these recommendations much deliberate thought before the study or whether their intentions had been stable for some time, it remains to be established whether participants' reported positive intentions will translate into actual behavior.

In conclusion, this study is an important step in increasing the understanding of dyadic processes in preventive screening and health. The findings provide an excellent basis for further research needed to shed light on communication processes and actual screening behavior.
